# From fear to fun: leveraging AI-powered videos in nursing education for disaster preparedness

**DOI:** 10.1186/s12912-026-05148-z

**Published:** 2026-08-04

**Authors:** Joanna W. Y. Yeung, Fiona W. K. Tang, H. Y. Cheng

**Affiliations:** The Nethersole School of Nursing, The Chinese University of Hong Kong, Hong Kong, HKSAR, China

**Keywords:** Disaster planning, Artificial intelligence, Nursing education, Curriculum

## Abstract

**Background:**

Disasters are increasingly prevalent nowadays, whether it is mass casualty incidents, pandemics, or the growing impact of climate change and conflicts. Nursing students should be prepared to be competent nurses, so that they can react safely and efficiently in the healthcare system. However, research on how advanced technology, such as artificial intelligence (AI)-powered videos, in disaster nursing education remains limited. Therefore, this study aims to investigate the impact of AI-powered animation videos on undergraduate nursing students’ knowledge and learning perception of disaster preparedness.

**Methods:**

This study adopted a quantitative design involving seventy-five undergraduate nursing students. A flipped classroom approach, grounded in the Cognitive Theory of Multimedia Learning, was implemented through the use of micro-modules with AI-powered videos and interactive mini-games. Students’ disaster preparedness was assessed by using a pretest-posttest approach, with the pretest administered one week before the micro-module and the posttest conducted at the end of the course.

**Results:**

Significant improvement in disaster preparedness was shown in the posttest compared with both the pretest and the prior cohort. Students reported feeling confident with self-directed learning (mean scores: 3.88 to 3.99) and satisfied with the content delivery (mean scores: 3.95 to 3.99) and expressed high overall satisfaction (mean scores: 3.93 to 4.01).

**Conclusion:**

The findings suggest that the integration of AI-powered videos into the flipped classroom curriculum was feasible and effective strategy for disaster education in nursing curricula. By presenting complex disaster preparedness concepts, in manageable sessions, the intervention facilitated significant improvements in students’ knowledge acquisition and fostered positive learning perceptions. These findings underscore the potential of AI powered instructional strategies to strengthen disaster preparedness among nursing students and support the development of a more resilient and practice ready healthcare workforce.

**Supplementary Information:**

The online version contains supplementary material available at https://doi.org/10.1186/s12912-026-05148-z.

## Introduction

The ongoing threat of disasters caused by climate change (e.g., droughts and hurricanes) and other large-scale crises, such as the COVID-19 pandemic, pose significant global challenges. These disasters negatively impact the physical and psychological health of individuals, families, and communities. Nurses are vital to frontline healthcare, helping the victims of disasters from the field to the emergency room and throughout communities. Skills such as triage and crisis communication can enhance the work of nurses globally. However, many nursing students identified the need for additional training for disaster preparedness in a recent study [1]. There is a critical need to evaluate and update the existing nursing curriculum to incorporate robust disaster preparedness competencies.

Traditional disaster education in nursing often relies on classroom lessons, simulations, and interdisciplinary and community partnerships [2]. Students’ clinical reasoning ability in disaster preparedness can be improved through the triage simulation and a structured debriefing [3]. However, these methods present several limitations, such as time constraints, the need to schedule learning spaces, and challenges with allocating a budget to the implementation of simulation programs [4]. These challenges also reduce the flexibility of content delivery [5]. In addition, there tends to be a lack of uniformity in the use of simulated patients in disaster simulations [6].

The flipped classroom is an innovative learning strategy that offers flexible and repeated training and is becoming increasingly significant in disaster education [7], particularly with the incorporation of digital tools such as training videos. This approach allows students to engage in active learning and develop their critical thinking skills during in-class activities [7]. Students can access the videos without time and physical space restriction, allowing them to learn at their own pace, and can replay the visual content multiple times, which reinforce self-learning [8].

Pre-recorded videos are the most common format used in the flipped classroom. When immediate knowledge retention was compared between nursing students who watched video-recorded lectures and those who attended live lectures, there was no significant difference [9]. Students disliked this teaching methodology because of the lack of interactive elements, which hampered knowledge retention and learning outcome maintenance [10]. There is thus a demand for more flexible, cost-effective, and engaging solutions for disaster education, and digital solutions such as AI-powered animated videos are gaining popularity with the adoption of the flipped classroom approach [11].

AI-powered videos generally categorized as: (1) instructor-based and (2) animation-based. In instructor-led AI videos, a script is fed into an AI system, which then generates a digital human instructor capable of delivering speech and facial expressions with a high degree of realism. In contrast, animation-based AI-powered videos are designed with software such as Vyond. When the software is provided a detailed script, a storyboard is automatically generated.

There are several advantages to using AI-powered videos as education tools. In terms of educational outcomes, AI-powered videos were shown to be effective for teaching foreign languages by improving students’ attitude, intention, and perceptions of the lesson’s usefulness [12] and increasing their knowledge retention [5] and learning motivation [13]. However, there was one study found no significant difference in learning outcomes between students who watched AI-powered videos, and the group who watched traditional instructor-made videos [14]. In all, this approach is flexible, allowing complex and customized topics to be covered [15]. Additionally, by providing more dynamic visuals to illustrate abstract concepts than traditional videos, it can reduce the cognitive load of lessons [5].

AI can be used to easily and cost-effectively convert presentations into a video format [16], allowing content to be tailored to diverse audiences and enhancing the reach and impact of educational programs [16, 17]. The use of AI also simplifies the creation and updating of visual content, requiring only simple script editing and video regeneration instead of cumbersome re-filming and editing [17].

The integration of AI in nursing education has demonstrated positive outcomes such as learning attitude, enhanced psychological effects and increased learning effectiveness [18]. However, research on the use of AI-powered videos in disaster nursing education is limited, particularly for the micro-module fitting in the flipped classroom. The effect of these videos on students’ learning outcomes and perceptions are still not well understood. Therefore, follow-up studies are essential to explore the effect of AI-powered videos and gain more comprehensive insights into how they influence educational outcomes and learning experience in this context. Also, it helps ensure that the integration of AI technology into disaster nursing education is both effective and supported by evidence.

## Methods

### Research aims

The aim of this study was to evaluate the effect of a disaster course incorporating AI-powered video micro-modules in increasing undergraduate nursing students’ knowledge and learning perception.

Research Question 1: Does the use of AI-powered video content improve nursing students’ knowledge and performance in tests following disaster management micro-modules?

Research Question 2: Does the use of AI-powered video content improve nursing students’ knowledge and performance in tests following disaster management micro-modules compared with a previous cohort?

Research Question 3: To what extent do nursing students perceive AI-powered video content provided in disaster management micro-modules support learning in terms of self-learning usefulness, delivery, and overall satisfaction?

### Study design

This study had a one-group pretest–posttest design aimed at evaluating the students’ knowledge of disaster nursing before and after participating in the micro-modules. This design is commonly used in educational research to assess the impact of interventions [19], especially since conducting randomized controlled trials in educational settings can be challenging [20]. Students’ learning perception was also measured at the end of study.

### Setting and participants

This study was conducted in a government-funded university in Hong Kong that offers a 5-year nursing program. The program studied was a three-unit course to introduce basic concepts of disaster management and disaster nursing to students. Data collection occurred between November to December 2024. Eighty-eight final-year students who enrolled in the disaster nursing course were invited to take part in this study. Based on an expected effect size 0.5, a standard deviation of differences of 10, a significance level of 0.05, and a power of 0.8, a minimum sample size of 32 participants was required to ensure sufficient power to detect significant differences between the pretest and posttest scores using paired t-tests. The inclusion criteria were students who were first-time enrollees in the course and agreed to participate. Those who had enrolled in the course previously or did not agree to participate were excluded. The students participated voluntarily and could leave the study at any time without any impact on their final grades. A total of 75 students participated in this study.

This study was a single-group intervention design. All eligible participants were assigned to the intervention condition with the AI-powered videos. To reduce potential bias associated with the absence of randomization, the study applied clear inclusion and exclusion criteria to ensure a consistent participant profile. Additionally, a standard intervention delivery of the AI-powered videos across all participants to maintain consistency in content and facilitation. Given the nature of the pedagogical intervention and single-group design, blinding either the participants or the facilitators of the study was not feasible.

### Theoretical framework

The development of the micro-modules was guided by Richard Mayer’s cognitive theory of multimedia learning (CTML) [21]. This theory is based on three key assumptions: (1) dual channels assumption, where information is processed through both auditory and visual pathways; (2) limited channel capacity assumption, recognizing that each channel has a finite ability to handle information; and (3) the active processing assumption, which involves learners engaging in selecting, organizing, and integrating information. CTML suggests that combining words with visuals leads to more effective learning than using text only. It describes five mental processes involved in learning: identifying key words and images, organizing them into meaningful verbal and visual structures, and connecting them with existing knowledge. This explains how the human mind processes multimedia information such as videos.

### Ethical considerations

This study was conducted with the approval of the Survey and Behavioural Research Ethics Committee of The Chinese University of Hong Kong. Informed consent was obtained. An information sheet was provided to all participants. The study was conducted in accordance with the principles of the Declaration of Helsinki.

### Intervention

#### Design of videos

##### Preparatory phase

AI-powered video-based micro-modules were used as a teaching method for flipped classroom learning in a disaster nursing course. We employed Vyond to generate five animation-based AI videos with engaging and explanatory content, including disaster management, risk assessment, emergency response, patient triage, and post-disaster recovery. Each video would be less than ten minutes.

Before the videos were designed, the project investigator (PI) interviewed four final year students to explore their baseline knowledge and learning needs regarding disaster nursing. The PI was an experienced nurse educator with rich disaster management experience developed the content of the micro-modules based on the students’ knowledge level in disaster preparedness in alignment with the learning outcomes of the course. Evidence shows that ensuring videos have clear objectives and structure is crucial for enhancing students’ understanding [16, 22]. The teaching team in this study reviewed the curriculum and selected topics suitable for delivery in an animated video format. The selection was based on the complexity of the content and the format. For example, conceptual ideas and practical content about triage procedure and disaster preparation were deemed suitable for video production.

#### Micro-module design: (1) Dual channel

##### Visual channel

In the CTML, students are said to be engaged and motivated by animation and narration. Therefore, the PI collaborated with the information technology department to plan the style of the videos and anticipate where pauses should be placed for discussions and short quizzes. The images and backgrounds were generated in several distinct visual styles. The content, which was based on common local disaster events, used images of commonly used rescue facilities, such as a local ambulance, to gain more familiarization to the local context. A casual animation approach was chosen as the main style for the videos in this micro-module, adopting colorful and animated characters and a vibrant graphic design based on the local context. The videos also included photos of local disaster events, ensuring that the contents were culturally relevant for the students.

##### Auditory channel

The videos were subtitled for easier comprehension, especially for the visual learner, rather than relying solely on the audio channel. The subtitles were in English, though other languages can be added for future use. AI was used to generate an animated character to ease the intensity of the learning experience and reduce the cognitive load, as well as voice-overs and captions.

#### Micro-module design: (2) Limited channel capacity

According to CTML, videos should have a concise and focused design and a maximum duration of 10 min to avoid overwhelming students with information. The videos for the micro-module were designed to align with the learning objectives to maximize their effectiveness [23]. The topics covered were disaster nursing principles, climate change and disaster, vulnerable populations in disaster I (older adults, pregnant women, children) and II (mentally ill and disabled persons), and the use of technology in disaster management, all of which are essential elements of the course.

#### Micro-module design: (3) Active learning

CTML explains that active learning involves mentally sorting through information- choosing what is relevant, organizing, and connecting it to what the learner already knows. Throughout the micro-modules, students participated in active learning tools such as interactive mini-games and discussions. For example, a game involving interactive decision-making points was used to test their knowledge of victim triage during a mass casualty incident. There were 2–3 mini-games within each video to gain learning engagement. Additionally, students were encouraged to participate in a discussion forum before proceeding to next stage. Teachers would post questions in the learning management system (i.e. Blackboard) to stimulate discussion for each video, enabling students to apply their knowledge during the discussion. Teachers would give feedback to clarify the key concepts.

#### Pilot test and final editing

In the final stage of production, the videos underwent several rounds of editing; adjustments were made to ensure that the content was accurate, and each scene was visually appealing and coherent with the narrative. The first drafts were reviewed by four students before the course launched to give feedback on different aspects, such as the speed, graphic design, and comprehensiveness. Their responses were generally positive, and some adjustments to the graphics were made based on their suggestion.

#### Delivery method

The videos were presented in flipped classroom micro-modules during the course, which lasted for three weeks in total. Students were able to view the videos from an online learning platform. Before seeing the videos, the students completed a pretest with ten multiple-choice questions; after viewing, they completed a posttest and learning perception questionnaire. After each video was played, a forum for discussion was provided in which the course teacher was available to answer questions. The students were allowed to view the videos multiple times without limitations. To arouse students’ interest in viewing the videos, a short preview trailer of around 1 min in length was created, and reminders were set in the online learning platform.

#### Measurement instruments

##### Knowledge acquisition

The research team prepared ten multiple-choice questions based on the information from various textbooks. Each question with four response choices was employed. Each correct answer scored one point. Thus, the more knowledgeable the student is, the higher their overall score. The same questions were administered in an alternative order in a posttest. A panel of multidisciplinary disaster experts validate these disaster preparedness knowledge questions. The expert panel included disaster rescue team members from aviation services, fire services, mountain search and rescue team. The overall Content Validity Index (CVI) was 0.92, indicating good content validity. Furthermore, the internal consistency of this instrument was assessed using Cronbach’s alpha, which was 0.612, indicating moderate reliability. Additionally, a statistically significant moderate positive correlation between pretest and posttest scores (*r* = 0.341, *p* = 0.003) was found, suggesting a moderate effect size.

##### Learning perception

A questionnaire developed by the principal investigator and reviewed by the research team, including nurse educators with experience in online and disaster education, to ensure item relevance and clarity (see additional information file 1). The questionnaire included 13 items rated on a 5-point Likert scale ranging from ‘strongly disagree’ (1) to ‘strongly agree’ (5) and assessed students’ perception of the usefulness of the self-learning content, mode of delivery, and overall satisfaction with the micro-module. A comment section was provided for qualitative feedback.

This questionnaire showed good to excellent internal consistency reliability with a Cronbach’s alpha of 0.927 for the total scale. The subscales for self-learning, delivery, and satisfaction had reliability scores of 0.862, 0.834, and 0.803, respectively. All exhibited good reliability, supporting the use of this questionnaire to evaluate students’ learning perception.

### Data collection and analysis

Statistical analysis was performed using the software program IBM SPSS 28 [24]. Since the study did not involve random assignment, an intention-to-treat (ITT) approach was not applicable.

For Research Question 1, student knowledge was measured using a pretest administered one week before the micro-module launch. Due to differences in individual learning pace, the posttest and learning perception questionnaire were completed at the end of the course. Paired t-tests were used to compare pretest and posttest scores, an appropriate method for analyzing changes within the same group over time. This statistical method is appropriate for comparing results from the same group at two different times.

For Research Question 2, while the course structure, content, and teachers were almost the same as for the previous cohort, except for the AI-powered animation video-based micro-modules, the groups had different sample sizes. Therefore, the final summative assessments of the current and previous cohort were compared using Welch’s t-test.

For Research Question 3, descriptive data and means and standard deviations (SD) were calculated to summarize the change in learning perception. Furthermore, the overall course evaluation of this cohort was compared with that of the previous cohort using Welch’s t-test.

Items with missing data or indicated as “not specified” were excluded from the respective analysis. The threshold level for noticeable values was set at > 5.0%.

## Results

### Demographic data

Out of 88 students who enrolled in the course, 13 students declined to join. 75 participated in the study, including 52 females (69.3%) and 23 males (30.7%), yielding a 85.2% response rate. The mean age was 22.3 (SD 0.79) years.

### Missing values

Missing data was minimal across all items, with less than 5% missing for each variable. As this fell below the predefined threshold for concern, no imputation or further adjustment was deemed necessary.

### Knowledge acquisition

Skewness and kurtosis were examined to confirm the normality of the data prior to conducting paired-samples *t*-tests to compare pretest and posttest scores across the 10 questions. The results demonstrated statistically significant improvements in eight of the 10 questions following the intervention. Effect size estimates indicated small to moderate learning gains (Cohen’s *dz* ≈ 0.34–0.62) for the significant items, suggesting a meaningful impact of the intervention on students’ knowledge acquisition. Details of the results were presented in Table 1.

**Table 1:** Disaster knowledge comparisons in pre-test vs. post-test (*p* < 0.05*; <0.001**)

Pre-test vs. Post-test	M	SD	Std. Error Mean	Cohen’s dz	95% Confidence Interval of the Difference		t	df	*p*-value
					Lower	Upper			
pre01 – post 01	-0.107	0.311	0.036	0.34	-0.178	-0.035	-2.973	74.000	0.004*
pre02 - post02	-0.053	0.432	0.050	0.12	-0.153	0.046	-1.070	74.000	0.288
pre03 - post03	-0.267	0.553	0.064	0.48	-0.394	-0.139	-4.173	74.000	< 0.001
pre04 - post04	-0.013	0.581	0.067	0.02	-0.147	0.120	-0.199	74.000	0.843
pre05 - post05	-0.147	0.356	0.041	0.41	-0.229	-0.065	-3.566	74.000	0.001**
pre06 - post06	-0.187	0.485	0.056	0.39	-0.298	-0.075	-3.335	74.000	0.001**
pre07 - post07	-0.280	0.452	0.052	0.62	-0.384	-0.176	-5.364	74.000	0.000**
pre08 - post08	-0.187	0.392	0.045	0.48	-0.277	-0.096	-4.121	74.000	0.000**
pre09 - post09	-0.173	0.381	0.044	0.45	-0.261	-0.086	-3.939	74.000	0.000**
pre10 - post10	-0.280	0.452	0.052	0.62	-0.384	-0.176	-5.364	74.000	0.000**

Overall, the findings demonstrate that the micro-module was effective in improving knowledge, as evidenced by the significant improvement in the posttest scores for the majority of questions. Furthermore, students in cohort AY2425 achieved significantly higher final examination scores (M = 17.03, SD = 1.95) compared with those in the previous cohort, AY2324 (M = 13.64, SD = 2.67). An independent-samples *t*-test confirmed a statistically significant difference between the two cohorts, with a mean difference of 3.39 points (95% CI [2.73, 4.06]), *t*(200) = 10.02, *p* < 0.001, indicating superior disaster knowledge performance among the AY2425 cohort (see Table 2).

**Table 2:** Comparison of Disaster knowledge across cohorts in the final exam

Cohort comparison	Mean difference	Std. Error Mean Difference	95% Confidence Interval of the Difference		t	df	*p*-value
			Lower	Upper			
AY2425 vs. AY 2324	3.394	0.326	2.726	4.062	10.021	200	< 0.001**

### Learning perception

The students’ learning perception was evaluated using a 5-point Likert scale, focusing on three subscales: self-learning, mode of delivery, and satisfaction. The results for each subscale are presented below (for details see Table 3).

**Table 3:** Learning perception after the use of AI-powered videos

Self-learning (SL)		Mean (SD)
SL01	The content provided in the micro-modules is relevant to my learning goals	3.96 (0.53)
SL02	The micro-module approach has facilitated me on active and independent learning.	3.89 (0.67)
SL03	The micro-modules have facilitated me to understand the topic in a simple and interesting way.	3.97 (0.61)
SL04	The micro-module approach is useful as pre-engagement BEFORE class teaching.	3.91 (0.57)
SL05	The micro-module approach is useful as topic review AFTER class teaching	3.88 (0.66)
SL06	The micro-modules have helped me understand the topics in a simple and engaging manner	3.99 (0.63)
Delivery		
D01	The structure of the micro-modules is well-organized and easy to follow.	3.99 (0.67)
D02	The delivery of the micro-modules keeps me engaged and interested.	3.97 (0.70)
D03	The time allocated for each micro-module is appropriate for the content covered.	3.96 (0.67)
D04	The multimedia elements (videos, graphics, etc.) enhance my understanding of the topics.	3.95 (0.71)
Satisfaction		
Sat01	I appreciate the flexibility of the micro-module format for my learning schedule.	4.01 (0.65)
Sat02	I feel more confident in the subject matter after completing the micro-modules.	3.93 (0.66)
Sat03	Overall, I am satisfied with my learning experience in these micro-modules.	3.97 (0.64)

#### Self-learning

The scores ranged from 3.88 (SD = 0.66) to 3.99 (SD = 0.63), indicating that the students generally felt confident in their ability to engage in self-directed learning.

#### Mode of delivery

The scores ranged from 3.95 (SD = 0.71) to 3.99 (SD = 0.67), indicating that the students were satisfied with how the content was delivered. The narrow range of scores and SDs indicate a uniformly positive reception.

#### Satisfaction

The scores ranged from 3.93 (SD = 0.66) to 4.01 (SD = 0.65), indicating a high level of satisfaction among students.

Overall, the analysis of learning perception subscales demonstrated that the students’ experience of this interactive micro-module was positive. They felt capable of self-learning, appreciated the mode of delivery, and were generally satisfied with the learning process. These findings suggest that the micro-module successfully provided the students with valuable and enjoyable learning experience.

Furthermore, the outcomes of the overall course evaluation (such as whether it was interesting and well-organized; enhanced knowledge acquisition; and enabled learning outcomes to be met) were similar between this cohort and previous cohort, except for the item “The course was stimulating” (Details see Table 4).

**Table 4:** Overall course evaluation comparisons for different cohorts

Category	AY2324 (*n* = 71)		AY2425 (*n* = 37)					
	M	SD	M	SD	Mean diff	t	df	*p*-value
The course was interesting	5.30	0.86	5.58	0.85	0.28	1.69	106	0.094
The course was stimulating	5.22	0.88	5.55	0.64	0.33	2.18	106	0.031*
The course enhanced my knowledge in this subject	5.28	0.80	5.55	0.67	0.27	1.87	106	0.064
The course was well-organized	5.22	0.77	5.45	0.69	0.23	1.62	106	0.109
Learning outcomes of the course were clear	5.23	0.79	5.47	0.66	0.24	1.69	106	0.095

## Discussion

### Knowledge acquisition

The findings of this study suggested that the AI-powered videos supported disaster knowledge acquisition among nursing students. These results are consistent with previous research finding that AI-powered animation videos enhanced learning outcomes [22, 25].

These studies were not directly related to disaster management, but due to the limited amount of relevant literature, key shared features were used to draw parallels with the knowledge gained in AI-powered video studies. For example, AI-powered videos with animated characters and tailored-made storylines can make complex, difficult, and abstract concepts easier to grasp. Moreover, including storytelling and dialogue in educational animations is crucial to maintaining student engagement [26], increasing their conceptual understanding of the content, and thus enabling effective knowledge acquisition [15].

Additionally, short and engaging videos break down difficult topics into more digestible segments, enhancing information retention, particularly for emotionally intense subjects [12] such as those covered in disaster education. According to CTML, intense subjects can limit the capacity of cognitive processing channels, which may hamper learning. By leveraging multimedia principles, the AI-powered videos may help manage cognitive load, even when the content is emotionally charged or complex.

Furthermore, the audio and visual functions of the videos can facilitate learning. Captions can help students who find language challenging or have varying levels of proficiency in understanding with the spoken content in the videos. Following along with the audio can reinforce the comprehension and retention of the taught material. In addition to captions, the use of storytelling and characters within AI-powered videos can further enhance accessibility, allowing students from diverse backgrounds to engage with the content more effectively and fostering a more inclusive learning environment [15].

In the present study, we also incorporated mini-games within the sessions of AI-powered videos to increase the interaction and check the understanding of students. Instant feedback was given after each game to enhance knowledge acquisition [5]. Given the blended learning approach based on the CTML used in the present study, we were unable to definitively conclude that the increase in knowledge was solely due to the AI-powered videos. The embedded mini-games and discussion forum may play a role in gaining knowledge. To determine the specific contribution of the videos to learning, future studies should provide videos as the sole intervention, without any additional interactive discussion forums or mini-games. Moreover, the duration of knowledge retention after viewing AI-powered videos for disaster education is uncertain; hence, researchers should continue to verify the educational impact of AI-powered videos in subsequent studies.

#### Learning perception

The observed acquisition of knowledge may be attributed to the students’ positive perception of learning [16]. The findings of this study suggested that the micro-module with AI-powered animated videos with interactive learning elements provided students with valuable and enjoyable learning experiences. The videos were successful tools for self-learning and resulted in high student satisfaction.

Students found that the video format facilitated self-learning. A similar result was found in the literature, with a study showing that nursing students repeatedly reviewed and analyzed the scenarios in training videos, leading to increased self-motivation and self-reflection [11]. In a study of computer science students provided an animated educational video, most participants reported that the animations helped them to understand the content, while 85% were “very satisfied” with the teaching [15].

In this study, the students reported that the use of multimedia was appropriate. Educators should understand how to use animations appropriately to avoid cognitive overload, which can affect the learning outcomes [12]. Proper animation design is essential. If animations are too complex or not well-integrated into the script or the storyboard of the video, they can overwhelm students’ cognitive capacities and hinder their ability to process and retain information, negatively affecting their learning outcomes.

In this study, the addition of the videos resulted in a course that had high student satisfaction and was perceived as more stimulating than indicated by the previous cohort. This may be due to the content being tailored to local disaster context and the use of multimedia. Using customized avatars and multimedia content is known to enable the creation of personalized educational experiences and facilitate learning [5, 17].

Moreover, comparable levels of satisfaction with AI-powered content were found in the literature, which were likely to be due to the clarity and appeal of the videos [5]; the use of animations and storytelling, which are helpful for understanding content [15]; and the high relevancy of the material to clinical practice [25].

However, the actual reasons for the increased satisfaction and interest in the education materials could not be assessed using quantitative data. This highlights a gap that should be addressed: namely, qualitative research should be conducted to better understand students’ subjective experiences. Additionally, a longitudinal study is suggested to investigate the students’ confidence over time. Lastly, as this study employed multiple embedded learning components including AI-powered videos, mini-games and discussion forum, with relatively lower weighting assigned to the latter two components, the findings should be interpreted as evidence of feasibility and potential educational benefit rather than definitive effectiveness of the AI-powered videos alone.

#### Implications

This study showed that AI-powered animated videos was associated with positive knowledge gains and favorable learning experiences in disaster education for nursing students. Given the increasing frequency and complexity of disasters worldwide, strengthening disaster preparedness among future nurses is a critical educational priority.

The findings offer valuable insights for nurse educators seeking to integrate AI technology into the disaster nursing curriculum to better prepare students for effective disaster response and resilience building.

In terms of the content, potential concerns such as bias and misinformation are associated with AI-powered material [5]. Therefore, educators must apply their professional knowledge to ensure that AI tools are used responsibly and that content is selected from reliable and evidence-based sources [14].

An AI-powered voice was used to present the content in the current study. However, teachers could provide voice-overs to potentially increase student engagement and bonding. This approach should be explored to understand its impact on learning outcomes, as it may enhance the personal connection felt toward the video material [13].

At the curriculum design level, AI-powered videos to enhance student knowledge and satisfaction can be part of the self-learning material in flipped classrooms. Such an approach allows students to acquire foundational disaster preparedness knowledge prior to face-to-face sessions, thereby freeing classroom time for scenario-based discussions and simulations.

Compared with traditional video production methods, AI-powered videos are less time-consuming and costly to prepare, allowing educators to focus more on content quality and less on production logistics [5]. Additionally, errors or updates in the video content about the disaster management can be easily implemented through script editing and video regeneration, rather than traditional refilming [12], ensuring that preparedness education remains current and responsive to evolving guidelines and risks.

However, adherence to ethical guidelines in AI content creation is crucial. In a 2024 study, only 24% of educators considered it necessary to disclose that a video was AI-powered, which raises ethical considerations [17]. Nonetheless, strict adherence to ethical guidelines in AI content creation is essential. Transparency regarding the use of AI-generated materials is particularly important to maintain trust and integrity in disaster education.

Looking ahead, the use of AI-powered videos has the potential to extend beyond nursing education to public health settings or primary and secondary education settings. It may be crucial for disseminating essential life skills such as disaster preparedness to the broader public. Additionally, adapting AI-powered videos for different languages and cultural contexts can improve educational inclusivity [15] and ensure that the content is accessible and effective across diverse populations [27]. Collaboration in reviewing the content of the videos, with frontline disaster experts can further enhance the relevance and authenticity of the learning materials, with alignment of real-world disaster response practices.

#### Limitations

This study has a few limitations that should be considered. First, the absence of a control group made it difficult to determine if the outcomes were directly caused by the intervention (AI-powered video micro-module) or other factors. Additionally, the study was confined to a single institution and not open access to the public, which limits the generalizability of the findings. The effect of AI-powered videos might vary significantly depending on the subject matter, student population, and educational context. To strengthen the evidence base, future studies should involve a range of institutions and learning environments. Another limitation is that the study did not provide insights into students’ knowledge retention and confidence levels over time; thus, the sustained impact of AI-powered videos on learning outcomes is unclear. Finally, a mixed-method approach would help to give a fuller picture of how students perceive their learning experience.

## Conclusion

With advancements in multimedia technology, AI-powered videos show promise as a feasible educational approach to support disaster preparedness in nursing education. As demonstrated in this study, these educational videos may help make complex disaster-related concepts more accessible, supporting diverse learning needs, and providing consistent foundational content.

When used as a part of blended or flipped learning approaches, AI-powered videos can complement classroom teaching and enhance students’ preparedness for disaster-related challenges. However, given the use of multiple embedded learning components and the study’s methodological limitations, the findings should be interpreted as indicative of feasibility and potential educational benefit rather than definitive effectiveness. Future research should employ more rigorous study designs, isolate the effects of AI-powered videos, and examine long-term knowledge retention and application across diverse educational and practice contexts.

## Supplementary Information

Below is the link to the electronic supplementary material.

Supplementary Material 1

## Data Availability

No datasets were generated or analysed during the current study.
