# Supplementary material for: From fear to fun: leveraging AI-powered videos in nursing education for disaster preparedness
Source: BMC Nurs. 2026 Aug 4;25:1038. doi: 10.1186/s12912-026-05148-z (PMC13721578; doi:10.1186/s12912-026-05148-z)
Supplement: Supplementary file 1 [file 12912_2026_5148_MOESM1_ESM.docx]

**Additional information**

**Questionnaire: Learning perception after the use of AI-powered videos**

|  |  | **Strongly disagree**  **1** | **Disagree**  **2** | **Neutral**  **3** | **Agree**  **4** | **Strongly agree**  **5** |
| --- | --- | --- | --- | --- | --- | --- |
|  | **Self-learning** |  |  |  |  |  |
| 1 | The content provided in the micro-modules is relevant to my learning goals |  |  |  |  |  |
| 2 | The micro-module approach has facilitated me on active and independent learning. |  |  |  |  |  |
| 3 | The micro-modules have facilitated me to understand the topic in a simple and interesting way. |  |  |  |  |  |
| 4 | The micro-module approach is useful as pre-engagement BEFORE class teaching. |  |  |  |  |  |
| 5 | The micro-module approach is useful as topic review AFTER class teaching |  |  |  |  |  |
| 6 | The micro-modules have helped me understand the topics in a simple and engaging manner |  |  |  |  |  |
|  | **Delivery** |  |  |  |  |  |
| 1 | The structure of the micro-modules is well-organized and easy to follow. |  |  |  |  |  |
| 2 | The delivery of the micro-modules keeps me engaged and interested. |  |  |  |  |  |
| 3 | The time allocated for each micro-module is appropriate for the content covered. |  |  |  |  |  |
| 4 | The multimedia elements (videos, graphics, etc.) enhance my understanding of the topics. |  |  |  |  |  |
|  | **Satisfaction** |  |  |  |  |  |
| 1 | I appreciate the flexibility of the micro-module format for my learning schedule. |  |  |  |  |  |
| 2 | I feel more confident in the subject matter after completing the micro-modules. |  |  |  |  |  |
| 3 | Overall, I am satisfied with my learning experience in these micro-modules. |  |  |  |  |  |
|  | **Comment:** | | | | | |

**Please rate 1-5 on the following parameters based on the learning experience. 1= Strongly Disagree and 5= Strongly agree**
